# Diphtheria-like Pseudomembranous *Corynebacterium striatum* Chronic Infection of Left Ventricular Assist Device Driveline Bridged to Heart Transplantation with Dalbavancin Treatment

**DOI:** 10.3390/reports8040208

**Published:** 2025-10-19

**Authors:** Tommaso Lupia, Marco Casarotto, Simone Mornese Pinna, Silvia Corcione, Alessandro Bondi, Massimo Boffini, Mauro Rinaldi, Francesco Giuseppe De Rosa

**Affiliations:** 1 Unit of Infectious Disease, University Hospital Città della Salute e della Scienza di Torino, 10100 Turin, Italy; 2 Department of Medical Sciences, Infectious Diseases, University of Turin, 10126 Turin, Italy; 3 Division of Geographic Medicine, Tufts University School of Medicine, Boston, MA 02111, USA; 4 Microbiology and Virology Unit, University Hospital Città della Salute e della Scienza di Torino, 10100 Turin, Italy; 5 Department of Surgical Sciences, Infectious Diseases, University of Turin, 10126 Turin, Italy

**Keywords:** *Corynebacterium*, left ventricular assist device, dalbavancin, heart transplantation

## Abstract

**Background and Clinical Significance**: *Corynebacterium striatum* is an emerging multidrug-resistant pathogen increasingly implicated in infections among immunocompromised patients and patients with indwelling medical devices. **Case Presentation**: We report the probable first case of pseudomembranous inflammation associated with *C. striatum* infection in a 53-year-old male with an implanted left ventricular assist device (LVAD) awaiting heart transplantation. The patient experienced recurrent episodes of *C. striatum* bacteremia despite multiple courses of targeted antibiotic therapy, including vancomycin, linezolid, tedizolid, teicoplanin, and dalbavancin. During urgent heart transplantation, pseudomembranous tissue surrounding the LVAD driveline was observed, and cultures confirmed *C. striatum* device infection. Histopathological analysis revealed necrotic elements and Gram-positive organisms consistent with pseudomembranous inflammation. **Conclusions**: The case describes the diagnosis and treatment of this rare infection, highlighting the pathogenic potential of *C. striatum*, its role in device-related infections, and the histopathological evidence of pseudomembrane formation.

## 1. Introduction and Clinical Significance

*Corynebacterium species* other than *Corynebacterium diphtheriae* may cause pseudomembranous inflammation during infection. The best-known examples are *C. ulcerans, C. pseudotuberculosis,* and *C. pseudodiphtheriticum,* which have been reported to exhibit clinical and histopathological features of pseudomembrane formation during the inflammatory process in cases of exudative pharyngitis, infective endocarditis, and lower respiratory tract infections [1,2,3].

Recently, in a lung transplant recipient, Charley and colleagues reported a case of *C. striatum* tracheobronchitis, characterized by a thick yellow adherent pseudomembrane on the bronchial surface observed during bronchoscopy [4]. These pseudomembranes were analyzed via histopathological samples containing necrotic elements with Gram-positive bacterial organisms [4]. *C. striatum* is a Gram-positive, ubiquitous colonizer of the human skin and of mucous membranes that, in recent years, has been increasingly implicated in infections of prosthetic devices or in immunocompromised individuals.

The ability to form biofilms may serve as an advantageous strategy for microorganisms to improve survival under stressful conditions, i.e., during host invasion or post-antibiotic treatment, as biofilm-associated cells exhibit significant resistance to human immune system components and to various antimicrobial agents [5]. Recently, Souza and colleagues reported data regarding biofilm formation on abiotic surfaces during *C. striatum* nosocomial outbreaks in a single-center study [6]. The results showed that *C. striatum* adhered to hydrophilic and hydrophobic abiotic surfaces [6]. Biofilm formation was confirmed by other authors, notably in multidrug-resistant strains of *C. striatum*; this formation ability correlates with its widespread transmission within the hospital environment [7,8].

Left ventricular assist devices (LVADs) are life-saving mechanical circulatory support systems for patients with advanced heart failure. Infections remain a major complication in this population, leading to significant morbidity and mortality [5]. *C. striatum* is also an emerging pathogen related to a high rate of drug-resistant strains [9] in this population.

Dalbavancin is a long-acting lipoglycopeptide with intravenous administration [10,11]. Its purpose is to suppress the development of cell walls with a large antimicrobial spectrum against Gram-positive pathogens such as *Staphylococcus aureus*, *Streptococcus pyogenes*, *Streptococcus agalactiae*, *Streptococcus anginosus*, *Corynebacterium* spp., and non-vancomycin-resistant *Enterococcus* species [10,11]. Dalbavancin has a half-life of about 346 h; according to the findings of pharmacokinetic studies, therapeutic concentrations of dalbavancin would be maintained in bone and articular tissue for a period of up to eight weeks if two doses of 1500 milligrams were administered one week apart [10,11]. Because of these pharmacokinetic and microbiological characteristics, dalbavancin is an appealing option for the treatment of Gram-positive infections that typically require prolonged antibiotic courses, such as osteomyelitis, septic arthritis, endovascular infections, infective endocarditis, and septic deep thrombophlebitis [10,11].

Our paper describes a pseudomembranous inflammation caused by *C. striatum* occurring alongside a driveline infection of an LVAD in a patient on a waiting list for heart transplantation, treated with a long course of dalbavancin as a bridge to transplant surgery.

## 2. Case Presentation

We report a case of a 53-year-old man suffering from heart failure with reduced ejection fraction of ischemic etiology who underwent LVAD implantation in September 2021 due to heart failure with refractory hypotension. From the cardiovascular point of view, the past medical history of our patient was characterized by arterial hypertension and a recent myocardial infarction in 2020 due to coronary artery disease, leading to a hypokinetic dilated cardiomyopathy. He was also a former smoker with a diagnosis of mild chronic obstructive pulmonary disease.

**Figure 1:**
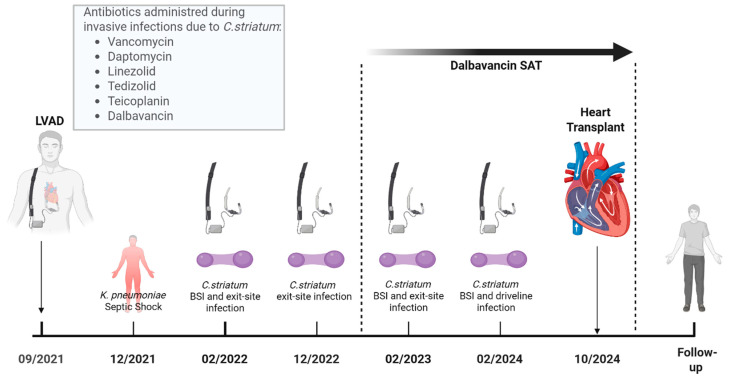
Timeline from LVAD implantation to heart transplantation. Abbreviations: LVAD: left ventricular assist devices; *K. pneumoniae*: *Klebsiella pneumoniae*; *C. striatum*: *Corynebacterium striatum*; SAT: suppressive antibiotic treatment; BSI: bloodstream infection.

**Figure 2:**
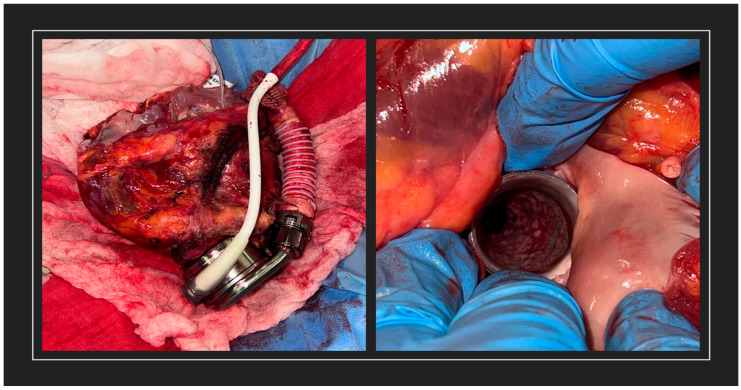
Pseudomembranous inflammation on LVAD driveline.

The postoperative course was complex as reported in Figure 1, marked by septic shock due to multi-susceptible *Klebsiella pneumoniae*, a cardiac tamponade, a pneumothorax, anuric acute kidney injury requiring dialysis, and a tracheostomy for the slow respiratory weaning after 32 days of intensive care unit. During the septic shock, a total-body computed tomography with contrast was performed to rule out driveline involvement or metastatic foci from bloodstream infection, with negative results. The patient was discharged in January 2022 and was enrolled in a rehabilitation program. In February 2022, following the appearance of a cough and increased inflammatory markers, he was readmitted and started empirical antibiotic therapy with ceftriaxone and azithromycin for a suspected chest X-ray consolidation in the left lower lung lobe. A respiratory film-array for virus and respiratory pathogens through nasal swab and urinary antigen was performed, with negative results. Blood cultures turned out positive for *Corynebacterium striatum*, confirmed on a culture from a swab of the VAD access site, and the patient was switched to intravenous vancomycin plus linezolid. A total-body computed tomography with contrast was performed to rule out driveline involvement or metastatic foci from bloodstream infection, with negative results. Intravenous (i.v.) vancomycin (15 mg/Kg every 12 h, after loading dose, adjusted weekly with therapeutic drug monitoring of vancomycin levels) and oral linezolid (600 mg every 12 h) were confirmed after that antibiogram showed susceptibility to vancomycin (MIC 0.5 µg/mL) and linezolid (MIC 0.38 µg/mL) and resistance to ciprofloxacin, clindamycin, gentamicin, penicillin G, rifampicin, and tetracycline. The patient completed 21 days of i.v. vancomycin. Oral linezolid was discontinued after ten days of treatment in combination with vancomycin due to a rapid reduction in platelet count. Blood cultures were cleared after 48 h of appropriate antibiotic treatment, and the patient was discharged with the recommendation of a 7-day course of oral tedizolid (200 mg every 24 h) to complete a four-week antibiotic regimen due to a suspected driveline-complicated infection. We decided to complete a four-week regimen of antibiotics despite not having confirmed driveline infection via CT, due to the multi-drug resistant pathogen being isolated, the confirmed exit site infection, the low sensibility of CT regarding driveline infections (without collections), and the risk of false positives in the case of positron emission tomography/computed tomography (PET/CT) not being performed due to the recent surgical event. In June 2022, the patient was re-hospitalized for hemoptysis. He also reported intermittent low-grade fever (37.7 °C) over the previous two weeks. The patient was started on piperacillin/tazobactam (4.5 g every 6 h) and intravenous vancomycin (15 mg/Kg every 12 h, after loading dose, adjusted weekly with therapeutic drug monitoring of vancomycin levels) for suspected pneumonia and chronic LVAD infection due to *C. striatum*. Bronchoalveolar lavage turned out negative, but blood cultures were again positive for *C. striatum*. Piperacillin/tazobactam was discontinued after five days, and the regimen was switched to teicoplanin (12 mg/Kg every 24 h, after loading dose) to complete 21 days of therapy. He was discharged with instructions to receive two 1500 mg IV doses of dalbavancin on days 0 and 14. Recurrent episodes of bacteremia due to *C. striatum* led to further hospitalizations and repeated antibiotic therapy from September 2022 to January 2024. During recurrences, CTs were performed to exclude driveline or complicated LVAD infections. Due to the negative results on CT regarding suspected driveline infection, in January 2024, a PET/CT was performed, which confirmed an uptake of contrast throughout the driveline. According to the rapid response to antibiotic treatment, no collections alongside the driveline and no surgical indications were given. The patient was then started on suppressive dalbavancin therapy with repeated administrations from February 2024 to October 2024 based on therapeutic drug monitoring, as reported in Figure 1. Despite targeted antibiotic management and infectious disease monitoring, the patient experienced multiple recurrences of *C. striatum* infection, suggesting the persistent colonization of the VAD access site and driveline. Initially, heart transplantation was delayed due to the high risk of surgical site infections or sepsis after the surgical event. Due to limited therapeutic options to control the chronic infection, heart transplantation was urgently demanded in October 2024. During the surgical procedure, a pseudomembranous inflammation was observed alongside the LVAD driveline, as shown in Figure 2, and multiple cultural samples turned positive for *C. striatum.*

The last dose of dalbavancin was administered the day after heart transplantation, and the patient was discharged after two weeks from surgical intervention. An immunosuppressive regimen with tacrolimus and mycophenolate mofetil was then started. Moreover, oral prednisone (0.2 mg/Kg) was started after heart transplantation and discontinued after six months post-intervention, and tacrolimus and mycophenolate mofetil were continued. After discharge, cardiologists and heart surgeons followed up with the patient monthly. As of now—twelve months after surgical transplantation—the patient has not reported any signs or symptoms of infection recurrence or transplant rejection.

Surgical illustrations show a pseudomembranous appearance of inflammation alongside the LVAD, which was found during heart transplantation.

## 3. Discussion

To our knowledge, this is the first described case of a pseudomembranous chronic infection of an LVAD driveline attributed to *C. striatum*. The genus *Corynebacterium* has around eighty published named species. *Corynebacterium diphtheriae, Corynebacterium ulcerans,* and *Corynebacterium pseudotuberculosis* are the only species known to be capable of producing diphtheria toxin (DT), a potent exotoxin that significantly contributes to pathogenicity [12]. The formation of diphtheria pseudomembrane involves DT production in the patient’s nasopharynx. *C. striatum* is currently considered a non-toxigenic species [12], but microbiological in vitro investigations indicate that the capacity to form biofilm on various abiotic surfaces is the most critical virulence factor [13]. *C. striatum* establishes colonies with similar efficiency on both hydrophobic and hydrophilic surfaces, including polystyrene ureters. The ability of *C. striatum* to form biofilm is likely enhanced in vivo due to the stimulatory influence of fibrinogen [13]. Biofilm formation increases bacterial resistance to immune system cells and antimicrobial drugs, which may explain the increasing incidence of catheter-associated bloodstream infections caused by *C. striatum* [13]. Microbial biofilms also play a crucial role in the development of endocarditis [13].

Theoretically, this significant biofilm formation during chronic device infections could explain our observation on the LVAD driveline during surgical debridement of the pseudomembranous inflammation, as seen in Figure 2. Few cases of pseudomembranous inflammation have been reported in association with *C. striatum* infections, both in device-related and in classical infections [14].

Recently, Zhu et al. reported a case of a bronchial stent infected by *C. striatum,* in which multiple necrotic pseudomembranes narrowing the lumen of the stent were seen during bronchoscopy [15]. As previously mentioned, Charley et al. published a similar observation in a lung transplant recipient in which a thick yellow adherent membrane was seen over the bronchus intermedius and tested positive for *C. striatum* [4].

Given the device-related infection and the high risk of biofilm formation, we initiated a prolonged and suppressive antibiotic treatment with dalbavancin, a new long-acting lipoglycopeptide. Several reports over the last ten years have assessed the use of dalbavancin in challenging off-label clinical scenarios, highlighting its innovative role in managing Gram-positive infections usually requiring long-term therapy [11], including previously reported experiences with prosthetic device infections due to *C. striatum* [16,17].

## 4. Conclusions

In conclusion, *C. striatum* is an emerging Gram-positive pathogen capable of early biofilm formation on device surfaces and pseudomembranous inflammatory tissue development during infection. Due to the high risk of antibiotic resistance among *C. striatum* strains—which limits available treatment options—prompt source control is pivotal in managing these infections, alongside the selection of antibiotics with anti-biofilm activity, high tissue penetration, and bactericidal activity. Dalbavancin may help in medical source control while waiting for definitive device removal.

## Data Availability

The datasets used and analyzed during the current work are available from the corresponding author upon reasonable request.
